# Observations on White Serum

**Published:** 1811-02

**Authors:** 


*For the Medical and Physical Journal.*

### *Observations on White Serum.*

WE have been favoured, by JOSHUA BROOKS, ESQ. (Theatre of Anatomy, Blenheim Street) with a portion of SERUM *of Human Blood*, which in appearance exactly resembles milk.

---

The

The blood, of which this *serum* made a part, was taken from a person suffering under *splenitis*, which was believed to arise from repeated attacks of intermittent fever. When drawn from the arm, it separated almost immediately into a white opake serum of the colour and consistence of *milk*, and a crassamentum (buffy and cupped) which floated in it. The patient was blooded five times. The first, second, and third bleedings exhibited the milky serum; the fourth and fifth had the serum of a natural colour and consistence, with a less sizy crassamentum.

The blood, from which this milky serum separated, was taken from the arm on the 24th of November ult.—and now, on the 25th of January, 1811, this serum has not undergone any change in its appearance and qualities, remains perfectly free from putridity, and looks like milk fresh from the cow.

The Gentleman, *Mr. Cullen*, Surgeon of Sheerness, from whose patient the blood was taken, and who transmitted the serum to Mr. Brooks, describes the bason of blood as precisely resembling a bason of milk, with the crassamentum floating in it.

The rare occurrence of this phenomenon, and the little that is at present known either concerning its cause or properties, or its connection with health and disease, induce us to lay before our readers the observations of that ingenious anatomist, Mr. Hewson, on the subject. Possibly this will be more acceptable, as we extract from a work become extremely scarce, and of a high price. Editor.

---

"Although the *serum* of human blood be naturally transparent, and a little yellowish, yet it is frequently found to have the appearance of whey, and sometimes to have white streaks swimming on its surface like a cream, and now and then to be as white as milk, whilst the *coagulum* is as red as usual. In all these three cases of whiteness, I have examined it in a microscope with a pretty large magnifier, and have found it to contain a number of very small globules, although naturally, when transparent, no globules can be observed in it, notwithstanding what has been affirmed by some authors. These globules differ from the red particles (improperly called globules) in their size, which is much smaller; and likewise in their shape, which is spherical, whilst the red particles are flat. They agree more with the globules of milk. I have compared them with those of woman's milk, and have found, that in the milk the globules are of different sizes, some being three or four times as large as others, and the smallest little more more than just visible, when viewed with a lens of $\frac{1}{27}$ of an inch focus, whilst those of the white *serum* are more regular, and are all of them about the size of the smallest globules of milk. Of white *serum* I have met with the following instances in books. In Tulpius, one instance,* in Morgagni, two,† in the Philosophical Transactions, some instances,‡ in Sckenckius's Observations, two cases are related from other authors. § I have likewise heard of the same appearance having been observed by the learned Sir John Pringle, Dr. Pitcairn, Dr. Hunter, Dr. Watson, Dr. Bromfield, Dr. Garthshore, and Dr. Fothergill of Northampton. And other instances have lately occurred to persons of my acquaintance who have favoured me with a short account of them.

" Mr. French, apothecary in St. Alban-street, having informed me, that he had some blood by him, taken from a woman the day before, whose serum was as white as milk; he favoured me with a small quantity of it for examination, and with it the following particulars of the case. ' Mary Rider, about twenty-five years of age, of a fresh complexion, and lusty, has not had her menses for these seven months. She discharges blood sometimes by vomiting, and sometimes by stool; complains of a pain in her left side, and in her stomach: she has an inclination to eat, but when she tries, she soon after loathes her food. She complains of great lassitude and sleepiness; her pulse is ninety-five in a minute. She has been bled twelve times within these six months, and every time the *serum* was as white as milk."

" Mr. Robertson, apothecary in Earl Street, acquainted me, that ' Mr. Herbert, a publican, of about thirty-five years of age, and corpulent, had been subject to a bleeding at the nose, to the piles, and to such profuse sweats in the night, as to be frequently obliged to change his shirt in the morning before he got out of bed, but that, for some time past, his sweats had ceased. That, on September the 23d, he was seized with a bleeding at his nose, which had been preceded by a pain in his head for two or three days. That his bleeding continued till he had lost about two pounds of blood, and then stopt; and that the serum of his blood was as white as milk. That at ten o'clock the same night, the hemorrage returned, and he lost a considerable quantity; nevertheless, it was thought proper to take sixteen ounces of blood from

* Tulp. Ob. l. 1. cap. 58.

† Morgagni, Ep. XLIX. Art. 22.

‡ Philosoph. Transact. No. 100, and 442.

§ Sckenckij Obs. lib. 3.

his his arm, during which evacuation he fainted, but his bleeding at the nose stopt. That the *serum* of this last blood was likewise very white. That on the 25th, in the morning, he again complained of a pain in his head, and about ten o'clock his nose began to bleed again ; but the *serum* now appeared no whiter than whey. That he continued to lose blood during most part of the night, so that it was supposed he could not lose less than two or three pounds, the serum all this time being a little whitish, but so little, that the bottom of the vessel in which it stood could now be seen through it. That his bleeding returned repeatedly till the third of October, when it entirely stopt, the *serum* having become more transparent towards the last."

"Mr. Eustace, apothecary in Jermyn-street, sent me a phial of white *serum* from one of his patients, by trade a butcher. 'This man,' he told me, 'was tall, of a strong make, a hard drinker, subject to puke every morning, took little food, sweated a good deal, but did not waste in his flesh. He was bled for a slight *asthma* to which he was subject, and of which he had always been relieved by bleeding. In other respects he was in a good state of health, so as to follow his business without much inconvenience."

"Besides these cases, my friend Mr. Lambert, surgeon at Newcastle upon Tyne, told me, 'that he had a patient some years ago with a violent rheumatic pain in his hip, whom he was obliged to bleed thrice, and every time his *serum* was as white as milk, but the *coagulum* of its natural colour. This gentleman,' Mr. Lambert adds, 'was a free liver, of a full make, but rather muscular than corpulent, and remarkable for being a great walker.'

"When I first saw this unusual colour of the *serum*, I was inclined to adopt the opinion of those who have attempted to explain it by the patient's being bled soon after a meal, or before the chyle was converted into blood. But afterwards, on considering the cases above related, I found this could by no means be the cause, as none of these patients had taken a sufficient quantity of food to occasion this appearance ; on the contrary, most of them had a bad appetite, and had taken remarkably little food, and were subject to vomitings. I therefore concluded it was owing to something else, and what confirmed me in this opinion was an observation I had repeatedly made in dissecting geese, whose *serum* I had frequently seen white, whilst their chyle was transparent ; although they had been killed only three or four hours after eating. And as the whiteness, in all the cases that I examined, was owing to a quantity of small globules like those of milk (which are known to be oily) I concluded that these in the human *serum*, when white, were oily likewise, and recollecting to have read somewhere of an experiment by which butter had been got from such human *serum*, I tried, by agitating some of it a little diluted, to separate its oil, or to churn it, but without success. I then inspissated some of it to dryness, and compared it with the natural *serum* of human blood prepared in the same way, and found it less tenacious, and much more inflammable; and when thus dried, its oil oozed out so much as to make the paper in which it was kept greasy. Another portion of this white *serum* being kept some days, putrified, and when putrid, it jellied as milk does when become sour; but it differed from milk, in being extremely fœtid.

"Now, as the white globules appear from these experiments to be of an oily nature, and as it is improbable, from these patients having taken little food, and from the transparency of the chyle in birds, that this whiteness of the *serum* should be owing to unassimulated chyle, accumulated in the blood-vessels; we must therefore believe it to be owing to some other cause. And as we know there is a considerable quantity of oil laid up in the cellular substance of animals, which is occasionally re-absorbed, is it not most probable that this curious appearance was, in the above-mentioned cases, owing to such a re-absorption? And as all these patients had symptoms of a *plethora*, and were relieved either by spontaneous hemorrhages, or by blood-letting, is it not probable, that, to whatever purpose the oil is applied in the body after it is re-absorbed from the cellular membrane, in these patients it had been re-absorbed faster than it was applied, and by that means was accumulated in their blood-vessels? This conjecture seems to be confirmed, from considering that in most of these cases the people were inclined to corpulency, and that two of them laboured under a stoppage of a natural evacuation.*

"Another conclusion which these observations lead us to is, that since the chyle of the birds which I dissected was not white, but transparent, at whatever time after eating it was examined, it follows, that the fat (in these animals at least) is

* Although it appears probable that the whiteness of the *serum* in the above-mentioned cases was not owing to the chyle, yet I would not conclude that the chyle does not in the human subject occasionally colour the *serum*. We frequently observe the *serum* of such people as are bled a few hours after a meal, a little turbid, like whey, which I believe may be owing to the chyle. But if the milk-like serum was occasioned by a full meal, it is likely we should oftener see it than we do.

not not merely the oily part of the chyle or of the food; but is a new substance, or a new combination of the principles or elements, which is made probably in the secretory organs of the adipose membrane: the form of oil being made use of by Nature in preference to any other for the nutritious substance of the body, from its being the least liable to putrefaction, and from its containing the greatest quantity of nourishment in the least bulk. This circumstance was clearly proved by my valuable and ingenious friend the late Dr. Stark, who, in a course of curious experiments, made by weighing himself after living for some time on different sorts of food, discovered that a less quantity of suet was sufficient to make up for the waste of his body, than of any other sort of ordinary food; and that, when compared with the lean part of meat, its nutritive power was, at least, as three to one.

"I may here add another circumstance that occurred to me when I first thought on this subject, which is, that since we believe the oil, or animal fat, is re-absorbed from the adipose membrane to serve for nourishment to the body; and as some of the patients (whose cases have been related above) could not take food, the re-absorption therefore of this oil might not be so much the cause, as the effects of the disorder under which they then laboured: or, in other words, that upon some defect in the digestive organs, the powers of nature drew from their magazines of oil in the adipose membrane, a supply of that fluid then perhaps necessary for the use of the body. In order to clear up this point, I thought it would be a satisfactory experiment, to compare the *serum* of the blood of animals at different periods after feeding them. For, if the re-absorption of the oil was merely to make up for the want of other food, or, if the *serum* was white merely from a greater quantity of oil being taken up in order to supply the wants of the body, then the *serum* ought to be whitest in the animal kept longest without food, or whose body was most in want. And as I had found that geese had very commonly this white *serum*, though their chyle was transparent, I chose to make the experiment on them. I therefore took two of them that were very hungry, and feeding both of them with oats, one I killed four hours after, when I knew a part of the oats were undigested; and upon examining the blood, I found the *serum* whitish, and full of small globules; on its being suffered to stand a little time, the white part ascended to the surface like a cream. The other was killed forty-eight hours after eating, when its stomach was found empty, and the *serum* of its blood quite transparent, and without any cream rising to the surface, or any appearance of small globules, when examined with the microscope.

X 2 Now,

Now, this experiment seemed to me decisive, and to point out clearly, that the whiteness of the *serum* was not occasioned merely by the body being in want of food, and therefore, drawing the oil from its magazines; because here the animal most in want of food had its *serum* least white; but was occasioned by the fat's being re-absorbed faster than it was used (from its place being supplied by the fresh chyle) and thence was accumulated in the blood-vessels, so as to give whiteness to the *serum*. And from the same observation it likewise appears probable, that the great re-absorption, and the accumulation of the fat in the vessels of the plethoric patients above-mentioned, was the cause of their want of appetite, and of their other complaints, and not the effect of them.

May not therefore a too great re-absorption of the fat, and its accumulation in the blood-vessels, be now admitted as the cause of one species of a *plethora?*

And may it not likewise be useful in some complains of the stomach, to attend to the whiteness of the *serum?* For, although fat be a substance little liable to disease, yet it may perhaps be sometimes so vitiated or may so incommode nature, that she may be obliged to take it up from her magazines, and to use it, or throw it out of the body. Whilst this is doing a sickness of the stomach, and want of appetite, may be indications of fulness; and therefore, instead of wanting remedies to strengthen the stomach, may require bleeding, and other evacuations.

